# COVID-19 Vaccines in Patients with Maintenance Hemodialysis

**DOI:** 10.3390/jpm11080789

**Published:** 2021-08-12

**Authors:** Cheng-Chieh Yen, Shang-Yi Lin, Szu-Chia Chen, Yi-Wen Chiu, Jer-Ming Chang, Shang-Jyh Hwang

**Affiliations:** 1 Division of Nephrology, Department of Internal Medicine, Ditmanson Medical Foundation, Chia-Yi Christian Hospital, Chia-Yi City 60002, Taiwan; u9001017@gmail.com; 2 Division of Nephrology, Chiayi Hospital, Ministry of Health and Welfare, Chia-Yi City 60096, Taiwan; 3 Division of Infectious Diseases, Department of Internal Medicine, Kaohsiung Medical University Hospital, Kaohsiung 80756, Taiwan; amoe616@gmail.com; 4 Faculty of Medicine, College of Medicine, Kaohsiung Medical University, Kaohsiung 80756, Taiwan; scarchenone@yahoo.com.tw (S.-C.C.); chiuyiwen@kmu.edu.tw (Y.-W.C.); jemich@kmu.edu.tw (J.-M.C.); 5 Division of Nephrology, Department of Internal Medicine, Kaohsiung Medical University Hospital, Kaohsiung Medical University, Kaohsiung 80756, Taiwan; 6 Department of Internal Medicine, Kaohsiung Municipal Siaogang Hospital, Kaohsiung Medical University, Kaohsiung 81267, Taiwan; 7 Institute of Population Health Sciences, National Health Research Institutes, Miaoli 35053, Taiwan

**Keywords:** anti-SARS-CoV-2 antibody, COVID-19 infection, COVID-19 vaccines, effectiveness, hemodialysis

## Abstract

The COVID-19 pandemic has infected more than 180 million people and caused more than 3.95 million deaths worldwide. In addition to personal hygiene, augmented cleaning, social distancing, and isolation, vaccine development and immunization are essential for this highly contagious disease. Patients with maintenance hemodialysis (MHD) have a greater risk of COVID-19 infection owing to their comorbidities, defective immunity, and repeated crowded in-center dialysis settings. However, many patients hesitate to get vaccinated because of their misunderstandings. The Efficacy of COVID-19 vaccination has been intensively discussed in the general population, whereas the data concerning the effectiveness of vaccination in MHD patients are relatively scanty. Nevertheless, those limited publications can provide some valuable information. Overall, lower and more delayed antibody responses following COVID-19 vaccination were observed in patients with MHD than in healthy controls in the settings of different populations, vaccines and dosage, definitions of the immune response, and antibody detection timepoints. Younger age, previous COVID-19 infection, and higher serum albumin level were positively associated with antibody formation, whereas older age and receiving immunosuppressive therapy were unfavorable factors. However, it remains uncertain between the elicited antibodies following vaccination and the genuine protection against COVID-19 infection. Patients with MHD should make their COVID-19 vaccination a priority in addition to other protective measures. More studies focusing on different vaccines, non-humoral immune responses, and risk-benefit analyses are warranted.

## 1. The COVID-19 Pandemic

In December 2019, a novel coronavirus was first detected in Wuhan, China. People who were infected by the virus manifested mostly as fever and pulmonary symptoms including dyspnea, productive cough, respiratory failure, and acute respiratory distress syndrome. Extrapulmonary involvements of the cardiovascular, gastrointestinal and, renal systems were also reported [1]. Owing to its rapid transmission, high contagiousness, and mortality, the World Health Organization (WHO) declared the outbreak a public health emergency of international concern on 30 January 2020. The virus was officially named severe acute respiratory syndrome coronavirus 2 (SARS-CoV-2) and the disease it caused was termed coronavirus disease 2019 (COVID-19) by the WHO on 11 February 2020. COVID-19 was then declared a pandemic on 11 March 2020. To date, more than 180 million people have been infected and 3.95 million people have died of COVID-19 [2].

Various treatment modalities for COVID-19 have been widely studied during the pandemic. Remdesivir can lessen respiratory symptoms and shorten the clinical course of hospitalization adult COVID-19 patients [3] and dexamethasone can improve the outcomes of hospitalized COVID-19 patients with oxygen dependence including mechanical ventilation [4]. However, the effectiveness of other medicines in the antiviral, anti-inflammatory, and immune-based fields remains uncertain. Since there are few effective treatments to control COVID-19, fundamental methods of stopping the spread of contagious diseases, including promoting personal hygiene and cleaning, enhancing decontamination, maintaining social distance, quarantine, isolation, and vaccine development and immunization are crucial.

## 2. COVID-19 in Hemodialysis Patients

End-stage renal disease (ESRD) is a condition defined as a renal function that cannot meet the homeostasis of waste, fluid, and electrolytes. According to the send edition of the *Global Kidney Health Atlas* published by the International Society of Nephrology in 2019, more than 2 million ESRD patients require dialysis or transplantation to stay alive, and hemodialysis is the most commonly used modality to treat ESRD [5]. Owing to their uremia and high burden of comorbidities including hypertension, diabetes, cardiovascular disease, and malignancy, patients with maintenance hemodialysis (MHD) have higher mortality than the general population. In addition, infectious diseases and sepsis are the major causes of hospitalization and mortality in this vulnerable population due to defects in innate and adaptive immunity [6,7].

Patients with MHD mostly receive their dialysis in the form of in-center hemodialysis and have to spend at least 10–15 h inside dialysis institutes with unavoidable proximity to other patients and medical personnel. Such a characteristic could increase the risk of accelerating the transmission of infectious diseases. Data from a national dialysis provider in the United States revealed that a crowded setting resulted in a 17-fold higher risk of SARS-CoV-2 infection [8]. The reported prevalence of COVID-19 among patients with MHD ranges from 5.3% to 36.2%, which is 5–16 times higher than the general population, and a mortality rate exceeding 20% [8,9,10,11].

## 3. Vaccination in Hemodialysis Patients

In spite of the defensive methods for COVID-19, patients with MHD remain at a higher risk of infection owing to their comorbidities, frequent invasive procedures, and crowded dialysis settings. Vaccination has been demonstrated to reduce the infection rate of some diseases including influenza, hepatitis B, and pneumococcal pneumonia in this cohort. However, many MHD patients hesitate to vaccinate owing to incomplete understanding and lack of trustworthy information. It results in a low rate of vaccination of the above-mentioned vaccines [12] and is similar in the case of the COVID-19 vaccines. Given that there are few effective medicines to treat COVID-19 at present and the high risk and possible mortality of the disease in MHD patients, vaccination is essential in addition to heightened sanitation, maintaining social distance, and cleaning. Major nephrology societies including the Internal Society of Nephrology, the American Society of Nephrology, and European Dialysis and Transplant Association—European Renal Association urge making COVID-19 vaccination a priority in this vulnerable population [13,14,15]. However, even in the United States, many MHD patients hesitate to receive a vaccination in consideration of the adverse effects [16].

## 4. COVID-19 Vaccines

Nearly 300 COVID-19 vaccine candidates have been developed to date and more than 100 are under clinical trials [17]. The brief summary below focuses on the vaccines which have received multinational emergency use authorization (EUA) and the preliminary results of their Phase 3 trials are listed in Table 1.

### 4.1. Inactivated Whole Virus Vaccines

An inactivated or killed whole virus vaccine comprises artificially cultured virus particles that do not have the ability to replicate and infect. The human immune system recognizes the entire virus particle as an immunogen following injection, which induces an adaptive immune response. There are three inactivated whole virus vaccines that have received multinational EUA for COVID-19 infection at present. CoronaVac has completed a Phase 2 trial of 950 adult participants and revealed a seroconversion rate (SCR) of 90.7% to 100% and neutralizing antibody geometric mean titers (GMTs) of 23.8 to 65.4 compared to placebo on Day 28 following vaccination [24,25]. BBIBP-CorV has completed a Phase 2 trial of 448 adult participants and revealed a SCR of 100% and GMTs of 14.7 to 282.7 in three vaccinated groups on Day 28 following vaccination [26]. BBV152 has completed a Phase 2 trial of 380 participants aged 12 to 65 years and revealed a SCR of 88% to 98.3% and GMTs of 100.9 to 197 in both groups receiving vaccination on Day 56 [27].

### 4.2. Viral Vector Vaccines

A viral vector, or deoxyribonucleic acid (DNA) vaccine, is a vaccine utilizing a nonpathogenic, usually replicative-deficient virus, to transport the gene of the immunogen into the human body. The modified virus will produce the immunogen to elicit an immune response following injection. With regards to COVID-19 viral vector vaccines, the ribonucleic acid (RNA) of spike protein, which is the pathogenic factor of SARS-CoV-2, is reverse transcribed to DNA and inserted into the genome of the chosen virus by genetic engineering. Nowadays, four viral vector vaccines have received multinational EUA for COVID-19 infection. AZD1222 was developed via modified chimpanzee adenovirus ChAdOx1. It has completed a Phase 2 trial of 23,848 adult participants and revealed an overall efficacy of 70.4% in participants receiving two doses of AZD1222 compared to placebo on Day 14 following vaccination [28]. Gam-COVID-Vac was developed via recombinant human adenovirus Type 26 and Type 5. It has completed a Phase 2 trial of 20 participants aged 18 to 60 years and revealed a SCR of 100% and GMTs of 45.95 and 49.25 in participants receiving vaccination on Day 42. In addition, significant cell-mediated responses were observed on Day 28 [29]. Ad26.COV2.S was developed via human adenovirus Serotype 26. It has completed a Phase 1–2a trial of 805 adult participants and revealed a SCR of 96% and GMTs of 288 to 488 in participants receiving the first dose of the vaccine on Day 57. A nearly three-fold increase in neutralizing antibody titers was observed following the second dose of the vaccine. In addition, cellular immunity was detected in 60% to 83% of the participants on Day 15 [30]. Ad5-nCoV, developed via recombinant human adenovirus Type 5, has completed a Phase 2 trial of 508 adult participants and revealed a SCR of 96% to 97% and GMTs of 18.3 to 19.5 on Day 28 in participants with a single injection [31].

### 4.3. Messenger RNA Vaccines

A messenger RNA (mRNA) vaccine is made by artificially synthesizing the mRNA of the immunogen to induce an immune response. Once the vaccine is injected, the immunogen is produced by interactions between its mRNA and ribosomes of the host cells [32]. With regards to COVID-19 mRNA vaccines, the mRNA of SARS-CoV-2 spike protein is manufactured and delivered in the form of lipid nanoparticles into the human body. Two mRNA vaccines of multinational EUA are available for COVID-19 infection now. BNT162b2 was developed using mRNA containing full-length spike protein. It has completed a Phase 2/3 trial of 43,448 participants aged over 16 years and revealed an efficacy of 95% in preventing COVID-19 7 days after vaccination [22]. mRNA-1273 was also developed using full-length spike protein mRNA. It has completed a Phase 2 trial of 600 adult participants and revealed a SCR of 100% and GMTs of 1686 to 1909 in participants receiving vaccination on Day 43 [33].

### 4.4. Protein Subunit Vaccines

A protein subunit vaccine utilizes protein molecule(s) as an immunogen to elicit an immune response following injection [34]. COVID-19 protein subunit vaccines are manufactured with the fragment(s) of the spike protein. They are free of hereditary substances and are thus distinct from vaccines made of an inactivated whole virus, reverse transcribed DNA or mRNA of the spike protein, and will not revert to the virulence of SARS-CoV-2. Two protein subunit vaccines of multinational EUA for COVID-19 infection are available currently. ZF2001 is a dimeric form of the SARS-CoV-2 receptor-binding domain (RBD). It has completed a Phase 2 trial of 900 participants aged 18 to 59 years and revealed a SCR of 93% to 97% and GMTs of 69.1 to 102.5 in participants who completed a three-dose-schedule after 14 days [35]. EpiVacCorona is a combination product of SARS-CoV-2 nucleocapsid protein, three fragments of the spike protein, and a bacterial maltose-binding protein designed and manufactured. It has completed a Phase 2 trial of 86 participants aged 18 to 60 years and revealed a SCR of 100% and GMT of 48.16 in participants 21 days following the second dose of the vaccine [36].

## 5. Effectiveness of COVID-19 Vaccines in Hemodialysis Patients

No patients with MHD have been recruited into efficacy trials of COVID-19 vaccines at present. In order to understand the effectiveness of COVID-19 vaccines in patients with MHD, we performed a literature search up to 03 July 2021 from the PubMed/MEDLINE electronic databases with the following search words: “SARS-CoV-2” or “COVID-19” and “vaccine” or “vaccination” and “hemodialysis”. Our search criteria were restricted to the English language. All relevant original articles, case series, or case reports were screened in our analysis based on their topics, impacts, and data availability. In total, 23 suitable studies were pooled in the review and divided into the category of “vaccine effectiveness between patients with MHD and healthy controls” and “characteristics following vaccination in patients with MHD”.

### 5.1. Comparison of Vaccine Effectiveness between Patients with MHD and Healthy Controls

Studies comparing the effectiveness following COVID-19 vaccination are summarized in Table 2. The results show that the antibody titers of patients with MHD were significantly lower than those of healthy controls, regardless of the antibody detecting methods used. It might partially result from the distinct age distribution of participants in both groups. Jahn et al. compared the titer of anti-spike protein glycoprotein IgG antibody two weeks after the second dose of BNT162b2 between 72 patients with MHD and 16 healthy healthcare workers and found that the seroresponses were significantly lower in MHD patients aged over 60 years [37]. Yanay et al. conducted a similar experiment of 127 MHD patients and 132 controls, and reported lower anti-spike antibody levels 21 to 35 days following two-dose BNT162b2 vaccinations in the dialysis group, particularly in those aged over 75 years [38]. Another experiment of 56 patients on MHD and 95 healthcare workers by Grupper et al. reported a significant inverse correlation between age and antibody levels in both groups 30 days following the second dose of BNT162b2 [39]. In addition, patients with MHD exhibited delayed humoral responses following COVID-19 vaccination. Goupil et al. reported that only 43% of 131 MHD patients had positive anti-RBD IgG levels, four weeks following a single dose of BNT162b2, compared to 95% of 20 controls [40]. Speer et al. also reported that 18% of 22 patients with dialysis had an early humoral response, compared to 93% of 46 controls, 17–22 days following the first dose of BNT162b2 [41]. Moreover, Simon et al. compared the adverse events of the participants following BNT162b2 vaccination. The local adverse effect such as pain following vaccination was reported in 10% to 20% of patients with MHD, whereas that was reported in 50% to 60% of healthy controls. The systemic adverse effect such as fatigue, headache or musculoskeletal pain was reported less than 10% of patients with MHD, whereas that was reported in 20% to 30% of healthy controls [42]. Danthu et al. analyzed the serial change of the anti-SARS-CoV-2 IgG antibodies among 78 MHD patients and seven healthy controls following two doses of BNT162b2. Serum albumin level and dialysis adequacy were reported positively correlated with humoral response, whereas the previous non-responsiveness of hepatitis B vaccination was negatively correlated with humoral response [43]. Rincon-Arevalo et al. conducted an experiment utilizing flow cytometry to observe the distribution of B and plasma cells specific to SARS-CoV-2 spike protein RBD. The RBD-specific B cells were identified mostly among subsets of the pre-switch and naïve B cells in 40 MHD patients, whereas mostly among subsets of the plasmablast or post-switch memory B cell in 35 healthy controls [44]. Taken together, these studies displayed the poor immune responses of patients with MHD following COVID-19 vaccination.

However, most previous studies have compared the effectiveness of vaccines according to the humoral response to SARS-CoV-2, and therefore, studies focusing on the cellular immune response following COVID-19 vaccination are warranted. On the other hand, most studies have shown the effectiveness of BNT162b2, although one study reported no significant difference in antibody response compared to AZD1222 [45]. More studies exploring the effectiveness of inactivated whole virus vaccines, viral vector vaccines, other mRNA vaccines, protein subunit vaccines, and mixed prime-boost schedules are also required.

### 5.2. Characteristics following Vaccination in Patients with MHD

The characteristics of patients with MHD following COVID-19 vaccination in the literature are summarized in Table 3. The reported age of these patients ranged from 62 to 76 years, and they were predominantly male (53% to 95%). Prior COVID-19 infection was diagnosed within 31% of the patients, and anti-spike protein IgG antibodies were detected in 34.7% to 95.4% following vaccination.

Among the factors associated with a favorable antibody response, previous COVID-19 infection was the most commonly reported. Chan et al. found that an anti-RBD IgG antibody response in MHD patients with prior COVID-19 infection was robust at one week following the first dose of mRNA-1273, whereas a robust response in those without prior COVID-19 infection did not occur until one week following the second dose of vaccine [46]. Billany et al. conducted an experiment of 94 patients with MHD and evaluated antibodies to S1 spike protein against COVID-19 28 days following a single dose of BNT162b2 or AZD1222. Their results showed that previous COVID-19 infection was an indicator of antibody detection [47]. Attias et al. studied the serial responses of anti-spike 1 IgG antibody following BNT162b2 vaccination in 69 patients with MHD. The rate of positive antibody was reported among those with a previous SARS-CoV-2 infection [48]. Younger age has also been associated with antibody response. A study of 101 vaccinated in-center hemodialysis patients by Torreggiani et al. reported that the age of neutralizing SARS-CoV-2 antibody responders was significantly lower than that of non-responders [49]. Frantzen et al. studied 244 in-center hemodialysis patients who received the two-dose BNT162b2 vaccination and observed that the younger patients were more likely to have an antibody response [50]. In addition, serum albumin level was also reported positively associated with antibody response. Agur et al. performed a prospective study of 122 MHD patients evaluating anti-spike SARS-CoV-2 IgG antibodies at an average of 36 days following two doses of BNT162b2. They observed that higher albumin levels, lower intravenous iron doses, and lesser body mass indexes were associated with higher antibody titers in the multivariate analysis [51]. Anand et al. assessed the anti-RBD IgG antibody responses among 610 MHD patients completing vaccination of mRNA-1273, BNT162b2, or Ad26.COV2.S. They found that MHD vintage and serum albumin levels were negatively associated with antibody response [52].

With regards to factors for a poor antibody response other than older age, Longlune et al. found that receiving immunosuppressants was an independent factor for antibody unresponsiveness one month following BNT162b2 vaccination in chronic dialysis patients [53]. Several other studies have also reported similar findings [47,50,54]. Lacson et al. further observed that non-responders after two doses of BNT162b2 or mRNA-1273 were more like to be female, have a shorter dialysis vintage, have received other vaccines, to be hospitalized within 14 days, and have congestive heart failure [54].

Different characters of patients, vaccine type and dosage, definitions of the humoral response, and timepoints of antibody detection may contribute to the variable results across the studies. BNT162b2 was shown to be effective in most previous studies, although the antibody response remained diverse among different vaccines [47,52,54]. Current studies have mostly evaluated the effectiveness of vaccines in the form of IgG titers. However, studies involving the cellular responses or human leukocyte antigen are sparse and inconclusive [55,56,57]. Further studies should be undertaken to enhance the understanding of immune mechanisms following COVID-19 vaccination in patients with MHD.

## 6. Conclusions

The effectiveness of COVID-19 vaccines was observed lesser and more delayed in patients with MHD than in healthy controls among different clinical settings. Less than half of the dialysis patients had positive antibody responses after receiving the first dose of the vaccine. Younger age, previous COVID-19 infection, and higher serum albumin level favored antibody formation, whereas older age and receiving immunosuppressive therapy were negatively associated with antibody formation. Evaluations of the effectiveness of vaccines have mostly focused on specific vaccines, and more studies focusing on different vaccine types, vaccine dosage, heterologous prime-boost schedules, other immune indicators, and risk-benefit analyses of vaccination are urgently needed. In addition, family members and healthcare workers involved with patients on MHD should be vaccinated against SARS-CoV-2, in order that transmission of the virus to those on MHD be minimized.

## Figures and Tables

**Table 1:** Progress of COVID-19 vaccines acquiring multinational emergency use authorizations.

Vaccine	Brand Name	Manufacturer	Type	WHO EUA	EUA Countries	Preliminary or Final Results of Phase 3 Trial(s)
CoronaVac	CoronaVac	Sinovac Biotech	Inactivated whole virus	Yes	26	434 participants enrolled; a SCR in neutralizing antibodies of 90% to 95.6% and an IFN-γ-based T cell response increase of 14.04 to 33.81 times on day 28 [18]
BBIBP-CorV		Sinopharm	Inactivated whole virus	Yes	45	40382 participants enrolled; efficacy of 72.8% to 78.1% in preventing COVID-19 [19]
BBV152	Covaxin	Bharat Biotech	Inactivated whole virus	No	9	25800 participants enrolled; efficacy of 80.6% in preventing asymptomatic COVID-19
AZD1222	Vaxzevria	Oxford University and AstraZeneca	Viral vector	Yes	102	32449 participants enrolled; efficacy of 79% and 100% in preventing symptomatic COVID-19 and severe disease and hospitalization, respectively
Gam-COVID-Vac	Sputnik V	Gamaleya National Research left	Viral vector	No	68	21977 participants enrolled; efficacy of 91.6% and 100% in preventing COVID-19 and moderate or severe COVID-19, respectively; a SCR of 98.25% and a GMT of 8996 in RBD-specific IgG on day 42; a higher levels of IFN-γ secretion on day 28 [20]
Ad26.COV2.S		Johnson & Johnson	Viral vector	Yes	44	44325 participants enrolled; efficacy of 66.1% and 85.4% in preventing moderate to severe-critical COVID-19 and severe–critical COVID-19, respectively [21]
Ad5-nCoV	Convidecia	CanSino Biologics	Viral vector	No	5	40000 participants planned; efficacy of 65.7% and 91% in preventing moderate COVID-19 and severe COVID-19, respectively
BNT162b2	Comirnaty	Pfizer and BioNTech	mRNA	Yes	85	43548 participants enrolled; efficacy of 95% in preventing COVID-19 [22]
mRNA-1273	Spikevax	Moderna	mRNA	Yes	49	30420 participants enrolled; efficacy of 94.1% in preventing COVID-19 [23]
ZF2001	Zifivax	Anhui Zhifei Longcom	Protein subunit	No	2	29000 participants enrolled
EpiVacCorona		Vector State Research left	Protein subunit	No	2	3000 participants planned

Abbreviations: GMT, geometric mean titer; IFN, interferon; mRNA, messenger ribonucleic acid; SCR, seroconversion rate.

**Table 2:** Comparison of effectiveness following COVID-19 vaccination between patients with MHD and healthy controls.

Country	Vaccine	MHDs	Controls	MHDs Ab	Controls Ab	Other Significant Findings	Ref.
Austria	BNT162b2	81	80	171 U/mL	2500 U/mL	MHDs exhibited lower local and systemic AE compared to controls (local: first dose: *p* = 0.006, second dose: *p* < 0.0001; systemic: first dose: *p* = 0.0005, second dose: *p* < 0.0001)	[42]
Canada	BNT162b2	154	40	9.5 RLU ^a^	124.5 RLU ^a^	Anti-RBD IgG level detection rate of 34% and 53% in older MHDs and younger MHDs, 19% and 48% in MHDs with immunosuppresants and MHDs without immunosuppresants following single-dose vaccination, respectively	[40]
France	BNT162b2	78	7	114 AU/mL	1082 AU/mL	Anti-SARS-CoV-2 IgG of 36, 113.5, and 209 AU/ml in non-responders, intermediate responders, and high responders to HBV vaccine, respectively	[43]
Germany	BNT162b2	72	16	366.5 AU/mL	800 AU/mL	Anti-SARS-CoV-2 IgG of 597, 414, 140, and 124 AU/ml in MHDs of 37–59, 60–69, 70–79, and 80–90 years, respectively	[37]
Germany	BNT162b2/AZD1222	23	14	1.6 AU/mL	73.1 AU/mL	Significantly higher IgG spike level (818.4 vs. 73.1 AU/ml) and IgM spike level (index: 0.86 vs. 0.34) in MHDs after COVID-19 infection than those of controls following single-dose vaccination	[45]
Germany	BNT162b2	22	46	6 ^b^	81 ^b^	Anti-S1 IgG index of 18% and 82% in MHDs following the first and the second dose, respectively	[41]
Germany	BNT162b2	40	35	60.3 ^c^	99.9	RBD-specific B cells identified mostly as naïve and pre-switch memory B cell in MHDs, whereas post-switch memory B cell and plasmablast identified mostly in controls	[44]
Israel	BNT162b2	127	132	116.5 AU/Ml ^d^	176.5 AU/mL	Anti-spike Ab of 99.5 and 122 AU/ml in MHDs less than 75 and more than 75 years, respectively	[38]
Israel	BNT162b2	56	95	2900 AU/mL	7401 AU/mL	Age positively associated with humoral response (OR, 1.22), whereas lymphocyte count negatively associated with humoral response (OR, 0.83)	[39]

Abbreviations: Ab, antibody; AE, adverse effect; AU, arbitrary unit; HBV, hepatitis B virus; MHD, maintenance hemodialysis; OR, odds ratio; RBD, receptor-binding domain; RLU, relative light units. ^a^ Antibody titer of patients with MHD was detected four weeks after vaccination, whereas that of controls was detected three weeks after vaccination. ^b^ The value of the anti-S1 IgG antibody test is expressed as an index: <1 was classified as negative, and ≥1 or higher as positive. ^c^ Forty MHD and 4 peritoneal dialysis patients were grouped together owing to no difference in age and vaccine response. ^d^ The study included 127 patients with MHD and 33 patients with peritoneal dialysis. The lowest anti-spike antibody level quartile group and the highest anti-spike antibody level quartile group did not differ in dialysis modality.

**Table 3:** Characteristics following COVID-19 vaccination in patients with MHD.

Country	Vaccine	MHDs	Age	Male	Prior COVID-19 Infection	Antibody Positivity	Favorable Factor(s) of Antibody Response	Ref.
France	BNT162b2	244	76	70%	13%	90.6%	Younger age	[50]
France	BNT162b2	101	69	59%	2%	34.7%	Younger age; lower comorbidity burden	[49]
France	BNT162b2	69	70	78%	19%	85.9%	Prior COVID-19 infection	[48]
France	BNT162b2	88	64 ^a^	69% ^a^	6%	84.1%		[53]
France	BNT162b2	10	71	N/A	N/A	88.9%		[56]
Israel	BNT162b2	122	72	66%	0%	93.4%	Younger age; higher albumin level; lower intravenous iron dose	[51]
Spain	BNT162b2/mRNA-1273	175	71	67%	N/A	95.4%	Higher lymphocytes; higher hemoglobin level	[57]
Spain	mRNA-1273	78	67	68%	N/A	94.9%		[58]
United Kingdom	BNT162b2/ADZ1222	94	62	60%	22%	79.8%	Younger age; prior COVID-19 infection	[47]
United States of America	mRNA-1273	61	70	95%	33%	95.1%	Prior COVID-19 infection	[46]
United States of America	BNT162b2/mRNA-1273	181	68 ^b^	53% ^b^	20% ^b^	89% ^b^	Male; longer dialysis vintage; prior COVID-19 infection	[54]
United States of America	BNT162b2/mRNA-1273/Ad26.COV2.S	610	N/A	N/A	15%	92.1%	Higher albumin level	[52]

Abbreviations: MHD, maintenance hemodialysis; N/A, not applicable. ^a^ The study included 88 patients with MHD and 24 patients with peritoneal dialysis. ^b^ The study included 181 patients with MHD and 5 patients with peritoneal dialysis.
